# Exploring the Associations of Afterschool Science Participation and Friendships with Science Identities

**DOI:** 10.1007/s11165-024-10173-6

**Published:** 2024-06-20

**Authors:** Patricia Wonch Hill, Grace M. Kelly, Julia McQuillan, Jorge Ledesma, Miranda Melson, G. Robin Gauthier

**Affiliations:** 1 Center for Math, Science, and Computer Education, University of Nebraska, Lincoln, NE USA; 2 Department of Sociology, University of Nebraska, Lincoln, NE USA; 3 Public Health, Brown University, Public Health, Providence, RI USA; 4 Department of Sociology, Northeastern University, Boston, MA USA; 5 IWK Health, Nova Scotia, Canada

**Keywords:** Science identity, Middle school, Peer influence, Afterschool science

## Abstract

Building on previous research that demonstrates the association of youth experiences in afterschool science and higher science identities, this paper presents a network study of 421 middle school students that examines afterschool science participation, friendship ties, and science identities. Participation in afterschool science clubs is associated with higher science identity, but the mechanisms and order of causality are unclear. Youth form friendships inside and outside of school, and peers may influence participation in afterschool activities, as empirical research on friendships shows that they are associated with youth interests. These peer interactions also have the potential to shape identity development during adolescence. In this study, we explore associations among youth participation in afterschool science clubs, peer friendship groups, and science identity. We find that youth who participate in afterschool science clubs have higher science identities than those who do not participate. Additionally, having friends in afterschool science clubs is associated with higher science identity, even among students who report not participating in clubs themselves. Results suggest that afterschool science clubs support youth science identities, even beyond those who directly participate.

## Introduction

Despite an increase in the number of graduates with degrees in science, technology, engineering, and math (STEM) fields in the last decade, scholars have concluded that the United States education system is not producing enough students to meet current demands (Wang & Degol, 2017). In addressing this STEM workforce shortage and need for a science-literate public, policymakers and researchers nationally have launched afterschool opportunities such as science programs, summer camps, and clubs to stimulate interest in science. There is evidence that these out-of-school opportunities to participate in science learning have the potential to produce meaningful results for youth who attend (Allen et al., 2019). Short-term outcomes include increased academic performance, engagement, enjoyment, and interest in science (Allen et al., 2019; Fallik et al., 2013). Afterschool science clubs, however, often compete with other activities, including sports, music, and art, and are not available to all youth (Afterschool Alliance, 2016; Hill et al., 2018a, 2018b; National Research Council, 2015). Research and theory indicate that, within practical constraints, youth choose activities based on identities that are most salient to them and that their participation in activities also influences identity development (Barber et al., 2005; Dabney et al., 2012). Because youth often self-select their out-of-school activities, it may be difficult to know if subsequent measures of interest and engagement reflect selection or causation.

The cultivation of science identity, or one’s identification with science, is consequential for persistence in science (Merolla et al., 2012; Merolla & Serpe, 2013; Riegle-Crumb et al., 2019). There is emerging evidence that stronger science and math identity have stronger associations with persistence in science and math fields than achievement in these subjects (Cribbs et al., 2015; Hill et al., 2018a, 2018b). Part of the complexity of self-selection into science club participation relates to how social context and peer groups shape personal identity and activity involvement (Barber et al., 2005; McPherson et al., 2001). Social identity theory (Tajfel et al., 2004) and empirical studies on the influence of peers on adolescent attitudes and behaviors (Brechwald & Prinstein, 2011) demonstrate that peer friendship group characteristics can have wide-ranging implications for youth in academic settings (Cook et al., 2007), and specifically for science identities (Lee, 2002, 2005). Using social network data from a United States middle school, the present study measures the association of the proportion of peers in a friendship group who attend afterschool science clubs and youth science identity, accounting for youth’s own participation in an afterschool science club.

## Literature Review

### Afterschool Activities and Youth Outcomes

Youth who participate in afterschool clubs report higher self-esteem, better grades, higher school belonging, higher graduation rates, increased likelihood of attending college, and broadened career aspirations (Durlak et al., 2010; Fredricks & Simpkins, 2012; Krishnamurthi et al., 2014; O’Donnell & Kirkner, 2014). Participation in afterschool clubs is also indirectly associated with outcomes such as reducing delinquency because they provide a positive environment for youth beyond the hours of the formal school day (Kort-Butler & Hagewen, 2011). Afterschool clubs can also endow useful, real-world skills that may connect to future careers. Compared to formal schooling, afterschool programs have less focus on academic performance, more focus on enjoying and exploring subjects, and provide additional opportunities for youth to make interpersonal connections with friends and mentors (Afterschool Alliance, 2014). Therefore, afterschool clubs have the potential to offer environments that are better suited for youth to explore and develop their interests and identities than formal classrooms (Barber et al., 2005). Social network studies show that the actions of friends matter for youth behaviors (Haynie, 2001) and therefore also matter for youth identities (McPherson et al., 2001). There is little information, however, about the potential for afterschool science clubs to have positive benefits for youth who are not directly participating in the clubs.

Based upon socioeconomic status and geographic location, some youth have more access to afterschool and extracurricular activities than other youth in the United States. Rural and urban youth in impoverished neighborhoods have less access to extracurricular activities than youth in suburban school districts (Afterschool Alliance, 2016; Hill et al., 2018a, 2018b; Sanderson & Richards, 2010). Even among youth who have opportunities for informal afterschool science, youth in poverty face barriers to participation. Compared to families with lower socioeconomic status, more affluent families can and often do invest time and financial resources to ensure that their children participate in extracurricular activities from young ages (e.g., zoo and museum visits, music lessons, art classes, and sports teams) (Lareau, 2011). Therefore, youth from families with more economic resources can accumulate more cultural, social, and scientific capital than their less affluent peers (Archer et al., 2015). Without public and private investments for youth with few economic resources, accumulating science capital will follow “the rich getting richer” pattern of those who already have more continuing to gain more through out-of-school science opportunities (DeWitt & Archer, 2017).

Inequalities in enrichment opportunities in childhood contribute to disparities in academic and social outcomes in early adulthood (Dufur et al., 2013). Archer et al. (Archer et al., 2012, 2015) developed a theory of “science capital” to illuminate how families cultivate science activities, identities, and future career aspirations based on their social location (e.g., social class, parent education, and occupational prestige). Access to fewer “extra” science opportunities (e.g., clubs, museums, zoos) means that some youth accumulate more science capital than others, and therefore youth vary in the opportunities and support that contribute to developing stronger identities as science kinds of people (i.e., science identities). Exposure to science language, ideas, ways of knowing, activities, and scientists themselves, all elements of science capital, not only increase science knowledge, but also opportunities to think of oneself as a science kind of person (Archer et al., 2012, 2015).

Cultural capital and science capital often require time and money (i.e., economic capital) because many science camps, museums, and zoos are not free and require flexible work schedules and transportation from adults. Accordingly, some government and non-profit informal science programs focus on supplying opportunities for youth from families that experience poverty.^1^ In the United States, 21st Century Community Learning Centers (CLCs) work to level economic inequities in access to capital by providing afterschool programming, enrichment, and educational services to youth in high-poverty areas. CLC goals are to “1) provide opportunities for academic enrichment; 2) offer students a broad array of additional services, programs, and activities; and 3) offer families of students served by community learning centers opportunities for active and meaningful engagement in their child’s education.” (U.S. Department of Education, 2018). These centers are primarily funded by the United States Department of Education and provide services for all fifty states, the District of Columbia, Virgin Islands, and Puerto Rico. Annual evaluations have shown that CLC program attendees show improvements in math and English grades, homework completion, class participation and behavior (U.S. Department of Education, 2018).

In addition to academic and behavioral improvement, CLCs may be particularly effective at leveling STEM disparities for youth from underrepresented racial/ethnic minority groups. Many inequalities in science capital exist *before* children begin school (Downey & Condron, 2016; von Hippel et al., 2018). Public school education can reduce inequalities in academic achievement, but there are limits to how much schools can make up for societal inequalities. Youth spend most of their time (up to 80%) outside of formal learning environments (Afterschool Alliance, 2020). Recognition of the limits of formal education has led to efforts to supply valuable science capital through informal learning within “summer programs, in libraries, museums, science centers, or at home or in the community” (Afterschool Alliance, 2018). Often youth from families with low socioeconomic status or those who live in rural areas do not have access to extracurricular science experiences. CLC afterschool clubs can give youth fun and valuable science enrichment that can reduce social-structural inequities. For youth in high poverty schools, science clubs are a key component of 21st CLCs, and therefore, can help youth overcome barriers to meaningful participation in science activities outside of classrooms.

### Science Identity

Afterschool science can be a particularly key component in developing a stronger science identity (Afterschool Alliance, 2013). Science identity is the ability to see oneself as a “science kind of person” or as a scientist. In a world where pervasive STEM stereotypes promote an exclusionary prototype of the typical scientist, being able to claim a science identity can be powerful (Shapiro & Williams, 2012; Starr, 2018). Factors such as academic achievement and science interest may not be as important for claiming a science identity as the ability to see oneself as a scientist and incorporate more STEM into one’s concept of self (Archer et al., 2012; Barton et al., 2013; Hill, McQuillan, Spiegel, et al., 2018a, 2018b; Packard & Nguyen, 2003). Instead of simply focusing on achievement or interest, science identity captures “how students are engaging in science and how that is related to who they think they are” (Brickhouse et al., 2000).

Informal science exploration in afterschool clubs can potentially strengthen youth science identities by demonstrating how science functions “in the real world” and exposing them to STEM career possibilities. Participation in afterschool science is an effective mechanism for increasing and maintaining student engagement in science (Dabney et al., 2012; Karp & Maloney, 2013; McCreedy & Dierking, 2017; Riedinger & Taylor, 2016; Sahin, 2013). Pursuing science beyond the hours of the school day can help youth feel like they are doing “real” science and increase the likelihood that youth develop stronger identities as science kinds of people (Allen & Noam, 2016). In contrast, many youth perceive that classroom science is out of touch with “real science” (Vincent-Ruz & Schunn, 2018; Wade-Jaimes et al., 2021; Zhai et al., 2014). Like science identity, science afterschool club participation has been positively linked to future outcomes such as continuing in STEM education and occupational aspirations (Cohen et al., 2019).

### Peer Influence

In adolescence, peers can have tremendous influence on each other’s behaviors, identity, and interests (Crosnoe, 2000; Crosnoe & McNeely, 2008; Ryan & Patrick, 2001). Just as students spend much of their time learning outside of a formal school day, peer influence is not limited to the academic environment. During the school day and outside of it, youth interact with peers in ways that shape their identities to such a degree that McPherson et al., (2001) argue that youth see themselves through their perceptions of how they think others see them. In their comprehensive review, Brechwald and Prinstein (2011) describe several mechanisms of peer socialization with implications for identity development. Therefore, like all identities, science identities are not formed in a social vacuum, but are constructed and maintained through social interactions.

Like popular notions of the power of peer pressure to shape adolescent behaviors, social network analysis shows how youth with similar characteristics are attracted to each other (i.e. homophily/selection) and socialize each other (for summaries see McPherson et al., 2001 and Brechwald & Prinstein, 2011). Peer influences spread through social networks and matter for behaviors such as smoking, alcohol, and drug use (Haynie, 2001). In addition to youth selecting friends whose behaviors and attitudes are similar to their own (i.e., homophily), friends’ attitudes and behaviors can converge over time through shared experiences and mutual socialization (Brechwald & Prinstein, 2011). A large body of research shows that social network dynamics can have implications for academic ambitions and success. At a school-wide level, students (even if not close friends) influence each other’s educational aspirations (Raabe et al., 2019). Additionally, grade point average is closely linked to friendship networks (Cook et al., 2007) and peer acceptance predicts academic progression (Lubbers et al., 2006). Even in studies that control for selection into homophilous friend groups, student peer groups can predict whether youth like and enjoy school and level of academic achievement (Ryan & Patrick, 2001).

There is also evidence that peers influence academic STEM preferences and advanced STEM course taking. Investigating student experiences within the context of science-related summer programs, Lee (2002) found that students with more relationships based upon science, medicine, and engineering interests also reported higher self-identification with and behaviors related to those fields. Others find that even controlling for youth science motivation, supportive friendship networks were associated with STEM career interest (Robnett & Leaper, 2013). Riegle-Crumb et al. (2006) found that the academic performance of friends was associated with an increased likelihood of enrolling in advanced calculus and physics courses, particularly among girls. A recent longitudinal study found that youth who participate in informal science programs are more likely to pursue STEM majors and careers, and that at least some of that association is likely due to interpersonal connections which help youth build STEM-related social networks and develop shared STEM identities (Habig et al., 2020).

Most studies of informal science participation, peer relationships, and associations with science only include those participating in formal or informal science specific programs (e.g. Brenner et al., 2014). Because such studies are longitudinal, they can use participant change over time to assess associations. The present study does not have longitudinal data but does include youth who do not directly participate in afterschool informal science clubs in the analyses and measures social network characteristics. Including social network characteristics is consistent with Brechwald and Prinstein’s (2011) acknowledgement that studies need to reflect how adolescent friendship dyadic relationships are “nested within larger networks, or cliques, or friendships” (p. 168). To model friendship group level characteristics and associations with science identity, this study investigates whether the proportion of one’s friends who participate in afterschool science clubs is associated with one’s own science identity, adjusting for whether or not youth themselves have been club members. The research questions are as follows.

## Research Questions

1. Does the current sample replicate past findings that youth who participate in afterschool science clubs have, on average, stronger science identities than youth who do not participate?
2. Are average science identities stronger among youth in friendship groups in which more members participate in afterschool science clubs than among those in groups with fewer participants in afterschool science clubs?

## Methods

### Survey Sample

The data collected for this study are from Wave I of the *Science Identity* study (collected in the Spring of 2014) (Hill et al., 2018a, 2018b). In order to model friendship networks within the school, we asked all youth enrolled in science courses at a middle school with a 21st Century Community Learning Center in a mid-sized Midwestern city to participate in an online survey. Due to the sensitive nature of nominating friends for a social network investigation, the school district determined that youth assent and affirmative parental consent were necessary for participation in the study. All parents or guardians were notified of the study with phone calls and emails with consent forms attached. The forms were available in four languages (English, Spanish, Vietnamese, and Arabic). In addition to parent consent, youth assent was obtained prior to youth participation in the survey.

Of the 663 youth enrolled at the middle school, sixty-seven percent (444) returned permission forms with parental consent. During the study, we asked youth various questions based on prior research regarding perceptions, attitudes, experiences, behaviors and social interactions about science (Archer et al., 2013; Hazari et al., 2010; Kier et al., 2014; Lee, 2002). We also asked youth to select their friends from a school roster and to answer questions about those friends. Research on adolescent friendship networks has shown that allowing five or more nominations is sufficient for accurately assessing adolescent social networks (Yang et al., 2009). In this study, we allowed students to list as many as 14 friends. This number was chosen to minimize potential student concerns about excluding friends, but still comply with time constraints in survey administration. To focus on group dynamics, youth who did not nominate any other students and were not nominated by any other students (deemed “isolates” in network science) were excluded from the current analyses (Wasserman & Faust, 1994). Thus, the analytic sample size for this study was 421.

The sample is demographically diverse. Only 36 percent identified as exclusively “white”; the remainder identified as another racialized minority group. Approximately 54 percent of the sample were girls. Of the students participating in the study, 29 percent were in 6th grade, 41 percent in 7th grade, and 30 percent in 8th grade. In addition, 66 percent of youth indicated that a parent or guardian had attended college. We did not identify participants’ socioeconomic levels due to constraints imposed by the school district and the Institutional Review Board. According to the school district, however, 78 percent of all students in the middle school are eligible for free/reduced meals, which indicates high levels of poverty (Nicholson et al., 2014; Snyder et al., 2019).

### Measures

*Science Identity* is a 3-item scale created by calculating the mean of available items that requires valid values for at least two of the items: (1) “How much do you think you are a science kind of person?” (1 = I am not a science kind of person at all, 2 = I am a little bit of a science kind of person, 3 = I am somewhat of a science kind of person, 4 = I am totally a science kind of person); (2) “How much, if at all, do you want to become a scientist?” (1 = Not at all, 2 = A little, 3 = Some, 4 = A lot); (3) “What kind of job do you want as an adult?” (1 = I want a job that does not use any science, 2 = I want a job that uses a little science, 3 = I want a job that uses some science, 4 = I want a job that uses a lot of science). The Cronbach’s alpha for this scale is 0.84. This measure is based on prior work on science identity and STEM career interest (DeWitt et al., 2016; Vincent-Ruz & Schunn, 2018).

Science Grades is measured by the question “What grades do you usually get in science?” (1 = Mostly A’s, 2 = Mostly A’s & B’s, 3 = Mostly B’s, 4 = A mix of A’s, B’s and C’s, Mostly B’s and C’s, 5 = Mostly C’s, 6 = Mostly below C’s). Responses were reverse-coded, and increments were shortened from 1 to 0.5 to create a scale from 1 to 4, where 1 indicates “Mostly below C’s” and 4 indicates “Mostly A’s”.

BIPOC is a dichotomous variable with 1 indicating race/ethnicity that is Black, Indigenous, or Person of Color and 0 indicating all other categories.

Parental College is measured by the item: “Did any of your parents/guardians go to college?” (0 = ”No” or “I don’t know”, 1 = “Yes”).

In-degree is a network measure that indicates how many nominations a student received. This measure is often used as an indicator of popularity. In our sample, this measure ranged from 0–20.

Out-degree is a network measure that indicates how many friendship nominations a youth made. We asked youth to nominate other youth in the school who they considered friends; they could select up to 14 friends. The range for this variable is 1–14.

Group Size was created using a Walktrap algorithm that created 27 friendship groups using network data (Pons & Latapy, 2005). Friendship group size indicates how many people are in a specific friendship group. This number ranges from 3–38.

*Proportion of Friends in Afterschool Science* is calculated for each youth by dividing the number of group members who participated in afterschool science (not including focal youth) divided by group size. This variable ranges from 0.0 to 0.8.

### Analytic Strategy

We used R to construct the school-wide network and to calculate in-degree and out-degree. Next, we used the Walktrap algorithm on the network data, which placed each participant in a mutually exclusive friendship group in which they had the most connections. Walktrap algorithms were designed to detect social communities—in our case, friendship groups—within the school (Pons & Latapy, 2005). The Walktrap method calculates groups by estimating the shortest path a person would have to “walk” to connect two nodes, compared to a “random walk” where the algorithm chooses each “step” at random (Smith et al., 2020). The Walktrap method is rooted in the idea that “nodes within communities are likely to be connected by shorter random walks” (Smith et al., 2020, p. 599). Youth (i.e., nodes) could be connected to others in a friendship group indirectly through shared friends or directly through explicit nominations. Thus, the algorithm identifies a friendship group as a community if the “walks” between the members are shorter than the walks from the members to people outside of the group.

We provide descriptive statistics for individual-level characteristics and friendship network characteristics. After calculating the individual variable descriptive statistics we also looked at group level differences between youth who reported participating in any afterschool science and those who did not. We provide means, standard deviations, and proportions between groups to explore group level differences.

Finally, we provide multivariate analysis to explore associations between the individual and friendship group level variables and science identity. Because the data collection was conducted with students in classrooms, the research design introduced clustering (Raudenbush & Bryk, 2002). Even though the clustering coefficient is small (ICC = 0.003), Clark (2008) finds that clustering can bias standard errors. We ran the models with and without accounting for clustering, and the conclusions are the same, yet to be conservative, we report the findings adjusted for clustering within classrooms. After adjusting for classroom clustering, the regression allows us to assess whether youth participation in afterschool science and proportion of friendship group participation in afterschool science are associated with science identity, controlling for individual, network, and group characteristics. We provide 95% confidence intervals to give an indication of the precision of the estimates based upon conventions in social science research, yet we do not refer to which coefficients are statistically significant at the 0.05 level because the sample does not meet the assumption of random assignment or random selection (Ioannidis, 2019).

## Results

**Fig. 1:**
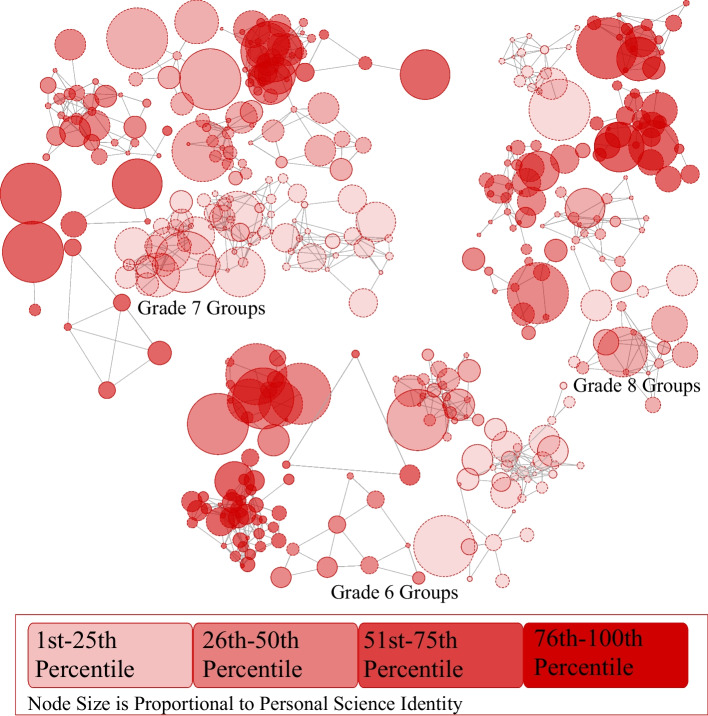
Network map of friendship group by grade level

Figure 1 shows the middle school youth network organized by friendship group and grade level. The Walktrap algorithm determined that there were 27 mutually exclusive friendship groups. Once the groups were determined, we calculated group-level differences in friendship peer group size and the proportion of friends in a group who participated in afterschool science clubs. These groups are indicated by the clusters of nodes (youth) connected by friendship nominations (lines). Each circle (i.e., node) represents one youth. The nodes in Figure 1 are sized according to the strength of the science identity of each youth: the larger the node, the stronger the science identity. Node color indicates the proportion of friends in a group who participated in an afterschool science club. The proportions were divided into quartiles that are represented by a color, with darker colors indicating a higher proportion of friends in a friendship group who reported that they had participated in an afterschool science club.

The distance between connected nodes was determined using the Fruchterman-Reingold algorithm, which is the default estimator in R, calculated using data from the entire network (Csardi & Nepusz, 2006). These coordinates were saved into a.csv file. Next, the Walktrap friendship groups were extracted and mapped using these coordinates. Figure 1 shows the connections only between youth within the 27 friendship groups, not the whole network map (Dalege et al., 2017). The distance between connected nodes (the lengths of the lines/edges) represents the strengths of the ties. Nodes that are connected and close together represent youth with closer ties than nodes/youth that are further apart. When two nodes appear to overlap it means they have larger nodes and/or a closer relationship. If the hypothesized direction of the association of proportion of friends who participated in clubs and strength of science identity exists, then we would expect to see that more of the larger circles (stronger science identities) would be darker red (more friends who participated in science clubs).

Table 1 shows the descriptive statistics for the total sample and tests for differences between youth who participate in afterschool science and youth who do not. Average science identity was 2.23 (SD = 0.79), a value that is near the mid-point of the range of values. Additionally, youth who participated in afterschool science had, on average, stronger science identities than youth who did not (M = 2.35 vs. M = 2.17). Approximately 54% of the students identified as girls, 61% of the sample were BIPOC, and there were more 7th grade students than 6th and 8th grade students (41% vs 29% each). There were very small differences, however, between those who did or did not participate in afterschool science for the proportion who are girls or BIPOC. Sixth grade youth were more likely to report attending afterschool science compared to 8th grade youth (37% vs. 24%). On average, youth reported relatively high grades in science (M = 3.2, SD = 0.77), but there were small differences in science grades for youth who participated in afterschool science and those who did not. Sixty-six percent of youth reported that a parent or guardian had attended college, and this was substantially higher for youth who participated in afterschool science compared to those who did not (75% vs. 61%).

**Table 1:** Descriptive statistics for individuals and peer groups, total and by afterschool science participation status (Yes/No)

	Descriptive Statistics				Group Differences			
	Total				No Afterschool Science (n = 281)		Any Afterschool Science (n = 140)	
	Mean/ Prop	SD	Min	Max	Mean/ Prop	SD	Mean/ Prop	SD
Individual Variables (*n* = 421)								
Science identity	2.23	0.79	1	4	2.17	0.80	2.35	0.77
Girl	0.54		0	1	0.52		0.56	
BIPOC	0.64		0	1	0.65		0.61	
Grade 6	0.29		0	1	0.25		0.37	
Grade 7	0.41		0	1	0.42		0.39	
Grade 8	0.29		0	1	0.32		0.24	
Grades in science class	3.20	0.77	1	4	3.19	0.78	3.22	0.75
Parent has some college	0.66		0	1	0.61		0.75	
In-degree	5.62	3.78	0	20	5.59	3.86	5.69	3.62
Out-degree	5.61	3.20	1	14	5.48	3.00	5.86	3.57
Peer Group Variables (n = 27)								
Peer group size	21.32	8.94	3	38	21.02	8.30	21.80	10.05
Proportion of friends who participated in afterschool science	0.33	0.47	0	1	0.30	0.14	0.40	0.13

We did not see large differences in friendship nominations (in-group or out-group) or peer group size between youth who reported attending afterschool science clubs and those who did not. On average, youth were nominated by 5.61 (SD = 3.78) peers and nominated 5.62 peers (SD = 3.20). Average peer group size was 21.32 (SD = 8.94), and almost exactly the same for both groups. Consistent with the principle of homophily, youth who participated in afterschool science clubs had, on average, more friends who participated in afterschool clubs than youth who did not participate (0.40 vs. 0.30). We cannot know if youth made friends in science clubs or joined clubs with their friends. Prior to conducting multivariate analysis, we explored bivariate associations among predictor variables to assess potential issues with multicollinearity. All bivariate correlations were below 0.42 (detailed results are available upon request). The conventional cut-off for problematic multicollinearity among independent variables is 0.60 or higher (Tabachnick et al., 2013), therefore the valu is below the level of concern for multicollinearity in the model.

Table 2 shows the results of the hierarchical OLS regression with individual science identity as the outcome, adjusted for nesting within science classrooms where the students took the survey. We also adjusted for overall network structure by controlling for in-degree and out-degree and for the variation in the size of the peer groups by including group size in the model. Grades in science, in-degree, out-degree, and group size were mean centered to adjust for multicollinearity and to make the constant more meaningful. Therefore, the constant indicates the average science identity for youth who have a zero value on all independent variables.

**Table 2:** Hierarchal OLS regression predicting strength of individual science identity (N = 421)

	Model 1				Model 2			
	β	SE	95% C.I		β	SE	95% C.I	
Girl (ref. boy)	**-0.160**	**0.078**	**-0.312**	**-0.007**	**-0.217**	**0.080**	**-0.373**	**-0.060**
BIPOC (ref. White)	-0.048	0.078	-0.200	0.105	-0.019	0.078	-0.172	0.133
Grade 7 (ref. 6)	0.064	0.088	-0.108	0.235	0.144	0.091	-0.035	0.323
Grade 8 (ref. 6)	-0.081	0.100	-0.277	0.115	0.045	0.109	-0.167	0.258
Parents have some college (ref. none/Idk)	0.122	0.076	-0.027	0.272	0.132	0.076	-0.017	0.280
Grades in Science	**0.327**	**0.048**	**0.232**	**0.421**	**0.333**	**0.048**	**0.239**	**0.426**
In-degree	-0.006	0.010	-0.026	0.014	-0.002	0.010	-0.022	0.018
Out-degree	0.006	0.012	-0.018	0.029	0.005	0.012	-0.018	0.029
Group Size	-0.008	0.005	-0.017	0.002	-0.008	0.005	-0.017	0.002
Participated in afterschool science (ref. no)	**0.165**	**0.110**	**0.015**	**0.315**	0.101	0.079	-0.054	0.256
Proportion of friends who participated in afterschool science					**0.834**	**0.295**	**0.255**	**1.413**
Constant	**2.205**	**0.110**	**1.990**	**2.420**	**1.884**	**0.157**	**1.575**	**2.192**
Model Fit Statistics								
AIC	951.160					945.271		
Wald Chi-Square	78.77					88.22		

In Model 1, 2.205 was the average value of science identity for 6th grade boys who are white; whose parents did not go to college; who have average grades in science, in-degree, out-degree, and group size; and who did not participate in afterschool science. In Model 1, girls, on average had weaker science identity (-0.160; CI -0.312 to -0.007) than boys, adjusted for the other variables. There was a positive association between grades in science with science identity (0.327, CI 0.232 to 0.421). After accounting for all other variables, youth who reported participating in afterschool science had stronger science identities than those who did not by about one fifth of a standard deviation (0.165, CI 0.015 to 0.315).

In Model 2, we added the measure of the proportion of friends who participated in afterschool science clubs. The constant now indicates the average strength of science identity for those in the reference groups who had the mean on all variables and had zero friends who participated in afterschool science clubs (a = 1.884). A mean science identity value of 1.88 is more than a third of a standard deviation lower (0.41) than the value for the previous model when proportion of friend group participation was not included. There was a positive association between the proportion of one’s friends who participated in afterschool science clubs and science identity, even after controlling for all other variables (B = 0.834, CI 0.255 to 1.413). After controlling for friend participation in afterschool science, youth participation in afterschool science was smaller than in the model without the measure of peer participation (from 0.165 to 0.101), suggesting that some of the association of club participation reflects peer dynamics and not only the content of the clubs. Science grades still had a positive and substantial association with science identity (B = 0.333, 0.239 to 0.426), or about 0.40 of a standard deviation. In addition, girls still had lower science identity than boys (B = -0.217, CI -0.373 to -0.060), or about a quarter of a standard deviation. Prior research indicates that peer associations with science outcomes may depend on gender (Gauthier et al., 2017), therefore we tested an interaction of gender by peer participation in afterschool science. The coefficient for the association of gender by proportion of peers who participate was very small, suggesting that the association does not depend upon gender (-0.215, CI -1.294 to 0.864) (not shown in table). 

## Discussion and Conclusion

Afterschool science experiences provide many youth opportunities to discover that they enjoy, are interested in, and see the relevance of various science topics (Krishnamurthi et al., 2014). Sociological theories of identity development emphasize the increasing salience of peer influences on youth identities and behaviors in adolescence (Crosnoe, 2000; Crosnoe & McNeely, 2008). Social network studies have advanced the ability of social scientists to explore peer associations with outcomes not only at the dyadic level, but also at the larger friendship group level (Brechwald & Prinstein, 2011). In this paper, our goal was to answer two questions about individual and friendship group participation in afterschool science clubs and strength of individual science identity. The answers to both questions were “yes”, both individual participation and proportion of friendship group participation is associated with stronger individual science identity. As far as we know, this is the first network study to find that, adjusted for one’s own participation or not in afterschool science clubs, the proportion of one’s friends who participate in afterschool science clubs is associated with stronger individual science identity.

There are important limitations to the current study. We used cross-sectional data from one school. Due to the cross-sectional nature of our data, we are unable to make claims about the associations with science identity that indicate directionality or causality. We can, however, assess if there is an association at all, and if it is worth designing future research to measure and model peer dynamics associated with science identities. Science identity processes are complex and emergent among adolescents, and research on science identities indicates feedback loops among youth and their peers over time. Expanding the sample and using longitudinal data could yield insight into these processes. Future research that follows youth over time (particularly as they add club participation and/or change friendship groups) will substantially strengthen or challenge the current findings. Additionally, including friendship information in studies that evaluate informal afterschool STEM clubs could help researchers identify potential ripple effects beyond direct involvement (Espelage et al., 2003; Sijtsema et al., 2010). With longitudinal data we could more accurately assess the potential value of investing in group-based opportunities. In a world in which remote and digital “learning” is increasingly an option for STEM opportunities, it is valuable to measure and model the potential costs and benefits of facilitating peer-group interactions and implications beyond direct knowledge gain such as identity development (McQuillan et al., 2023).

While the results of this study is consistent with prior research on science identity, prior research also indicates that science identities intersect in disparate ways for some youth who belong to groups historically marginalized in STEM (e.g., racial/ethnic minorities, those with lower socioeconomic status, those who are neurodivergent, and/or disabled) (Hill et al., 2018a, 2018b; National Science Foundation, 2017; Wong, 2016). Future studies should further explore whether the relationship between peer afterschool science participation and science identity differs by peer characteristics such as gender, race/ethnicity, or social class. For example, Raabe et al. (2019) found that girls specifically were more likely to maintain STEM preferences when they were surrounded by same sex peers who rated STEM subjects highly. Furthermore, in a study on middle school social networks and STEM attitudes, researchers found that middle school boys and girls were more likely to identify their male peers as a science kind of person than they were their female peers (Gauthier et al., 2017), therefore more effort may be required to increase science identities among girls.

The findings in this study are important for several reasons. As described above, youth vary in science capital based upon the resources in their homes. Youth with lower science capital likely also have demands, such as caring for siblings or elderly relatives, or transportation restrictions that inhibit direct involvement in clubs. Additionally, few schools have the resources or volunteers to accommodate all youth in a school. Only a fraction of youth can consistently participate in any one club (generally including fewer than 20 participants). This research supports the idea that there are potential positive impacts of afterschool clubs beyond the participating students (i.e., a small number of participating students can influence non-participating friends and the wider school). Understanding the extent to which 21st Century Learning Communities can provide benefits that go beyond direct participants is important as we consider future investments in these public programs in the U.S. Finally, the results of the current study support prior studies on the relevance of peers and identities for important social, educational, and career outcomes and extend the application of peer social identity theories to peer influences on science identities.

## Data Availability

The data for this study is not publicly available.

## Footnotes

1 High poverty or Title I schools in the United States are defined as schools where greater than 50% of students qualify for free and reduced lunch (Snyder et al., 2019). Free and reduced lunch qualifications vary from state to state. For this school, families who make less than 130% of the federal poverty rate qualify for free lunch. This is approximately $36,000 USD for a family a four (Nicholson et al., 2014).
